# From ‘I won’t protect you’ to ‘I will’: authoritarian protectionism and the end of neoliberal hegemony

**DOI:** 10.1057/s41311-023-00447-7

**Published:** 2023-03-21

**Authors:** Luke Cooper

**Affiliations:** London School of Economics and Political Science (LSE IDEAS), London, UK

**Keywords:** Democracy, Authoritarianism, Neoliberalism, Hegemony, Gramsci

## Abstract

The following article represents the author response to the roundtable on Authoritarian Contagion; the Global Threat to Democracy (Bristol University Press, 2021).

I would like to thank the contributors for taking time to engage with the ideas I put forward in *Authoritarian Contagion* in their challenging and intellectually rich pieces for the forum. In this reply, I respond to their points through several steps. First, I consider the contrast between my favoured analytical frame of ‘authoritarian protectionism’ and twentieth-century fascism in light of Russia’s war against Ukraine. Second, I elaborate the difference between classical neoliberalism and authoritarian protectionism as distinct hegemonic paradigms, building on Priya Chacko’s forum contribution and its emphasis on extra-economic exploitation. Third, I offer an alternative account of the Eurozone crisis to the one put forward in the forum by Richard Saull and consider how this impacts analysis of the global pattern of authoritarianisation. Lastly, I critically interrogate Reijer Hendrikse’s argument that both China and the USA should be read as converging on a shared model of ‘corporatist regime’.

## Putin’s revanchism and the authoritarian contagion

*Authoritarian Contagion* draws on a Gramscian framework to cast the new authoritarianism as an attempt by blocs—usually, but not always ‘insurgent’, anti-system forces—to win leadership in society through a process of ‘mass creation’ (Gramsci 1971, p. 341; see also Cooper 2021a, p. 20). For Antonio Gramsci, hegemony-seeking groups, whether progressive or regressive in their political complexion, will not win leadership positions in society without grounding their ideas and vision in existing underlying cultural sentiments in the population (ibid). The fascism of Gramsci’s era was such a ‘hegemonic project’. It linked together a harrowing ‘revolutionary’ vision (Paxton 2004, pp. 9–14; see also Griffin 1991) with everyday notions of ethnic grievance, xenophobia, patriotism and national security. This was reminiscent of, and partially modelled on (Saull 2015; Saull et al. 2015; and on the German case see Baranowski 2011), late eighteenth- and nineteenth-century European imperialisms that synergised modern and traditional themes (Chryssogelos 2015) to pursue the ‘civilisational’ reshaping of the world through empire. The oft-cited tenets of inter-war fascism, such as revanchist imperialism, white supremacy, popular mobilisation, state-managed capitalism and the cult of leadership (Eatwell 2003; Mazower 2000, chapter 1), were crafted to a historical narrative that depicted this transformation as an unavoidable task, necessary to save the nation and achieve true salvation.

Against this overarching historical backdrop and legacy, the authoritarian turn of the twenty-first century, for the most part, does not seek the degenerate ‘utopia’ (Kallis 2004) associated with classical fascism (Cooper 2021a, p. 129). China, as I argue in the book, is an unusual case, because Xi Jinping’s use of ethnonationalism and aggressive power centralisation is an example of an authoritarian development within a historically stable one-party dictatorship. However, even in this example, which comes close to classical fascism, the hegemonic strategy is status-quo orientated, a cautiousness that sets contemporary Chinese communism apart from the destabilising mobilisations of the Mao-era. Unlike Maoism, Xiism above all prioritises preservation. It seeks to sustain the institutions of one-party rule over the long term by framing them as the only vehicle able to secure the interests of the Han Chinese.

Since the publication of the book—and in a shock to many analysts, the present author included—Vladimir Putin’s invasion of Ukraine appears to provide a bleak affirmation of the perspective of those scholars (e.g. Faulkner 2017; Snegovaya 2017; Snyder 2022, 2017; Stanley 2018) that insisted contemporary authoritarianism was continuous with historical fascism. The difference between my analysis and these approaches may not, however, be as great as this suggests. As noted, I argued that authoritarian protectionism is anti-utopian in design. Xiism and Putinism constitute ideologies defined by a struggle for greatness in the existing world—not the creation of an entirely new order (Cooper 2021a, p. 61). Timothy Snyder has discussed this difference as a transition from a politics of *inevitability* to one of *eternity*; in the former genocidal violence becomes justified by a stadial account of history, but in the latter the future is displaced by an eternal present (Snyder 2018). The conjuring of threats to this order provides a veil of justification for the autocratic centralisation of power (ibid).

In my account, authoritarian protectionism follows a three-step logic, which operates like Snyder argues, in an ‘eternal present’, not the pursuit of a new stage of history: first, the ethnically defined nation is established as the core of all political reasoning; second, this entity is presumed to have partisan interests fundamentally opposed to those outside the in-group; and third, profound civilisational threats are conjured that allegedly risk the ‘certainties’ of the people. The *claim to protection* is the critical device across these stages.^1^ Owing to the alleged threats facing the 'insiders' attacks on democracy are suddenly cast as necessary and legitimate (Svolik 2019). Importantly, the primary agent of this vision is therefore not the abstract market individual of neoliberalism, nor the class-based solidarities of socialism, but an ethno-nation facing a range of grave threats. Unlike in classical fascism this agent is kept, for the most part, a passive subject of the claim to protection, rather than an active, mobilised street force.^2^

Putin’s now infamous essay of the summer of 2021, ‘On the Historical Unity of Russians and Ukrainians’, followed the logics of authoritarian protectionism closely. ‘Russians and Ukrainians’, he argued, are ‘one people—a single whole’ (Putin 2021). In frequently Orwellian terms (especially when read in the context of the subsequent invasion), the essay assimilated Ukrainian identity into a monolithic pan-Russian one. He decreed that Ukraine does not have a legitimate existence as an entity independent of, and separate from, Russia. Ukrainian statehood was held to be an invention of the Soviet Union and its ‘localisation’ policy*.* This imposed a Ukrainian identity on those that would have preferred to remain Russian and created a ‘dangerous time bomb’ that ‘exploded’ with the fall of the Soviet Union (ibid). The loss of the communist party meant no force was able to uphold the unity of Ukrainians and Russians. In this warped worldview, the invasion ‘corrects’ this error and restores ‘harmony’.

Putin wilfully ignores Ukraine’s pre-Soviet history. He defends the most imperialist elements of the Soviet Union (famously describing its collapse as ‘the greatest geopolitical catastrophe of the century’), while condemning its token commitments to self-determination. After all, the Ukrainian Soviet Socialist Republic had its own flag and a seat at the United Nations, which saw the state twice serve terms on the Security Council (1948–49 and 1984–85). For Putin, by contrast, *any* assertion of Ukraine’s independent selfhood can only be indicative of a malformity—a claim that has some parallels to Xi Jinping’s campaign of cultural genocide against the Uyghur people in Xinjiang. Dangerous and foreign threats—in the latter the so-called ‘sick thinking’ of Islam (cited in Raza 2019, p. 497) and, in the former a ‘neo-Nazi’ ideology subjecting Ukraine to the foreign domination of the West—are held to require ‘cleansing’, i.e. violent assimilation. Nonetheless, Putin’s willingness to abandon any pretence of legal justification, and utilise openly imperialist arguments, goes further than most of his global co-thinkers. In a manner that encapsulates what Ruth Wodak calls in this forum ‘the shameless normalisation of previously tabooed agendas’ (see also Wodak 2020), Putin has compared his war to the conquests of Peter the Great (Guardian News 2022). In doing so, he effectively discarded the UN Charter’s touchstone principles of sovereign equality and territorial integrity. Kremlin-aligned outriders have made similarly stark imperialist and even genocidal statements. In one example, state media agency, RIA Novosti, called for a campaign of repression and liquidation in Ukraine under the absolute control of the Russian state, explicitly rejecting any notion of Ukrainian sovereignty. This ‘denazification’ would, they said, ‘inevitably also be de-Ukrainisation’, adding that ‘“Ukraine” cannot be kept as a title of any fully denazified state entity on the territory liberated from the Nazi regime’ (Sergeytsev 2022). This raw fascism, veiled in ‘anti-Nazi’ discourse, now defines the Kremlin’s view of the world.

## Extra-economic exploitation and shifting hegemonic paradigms

Several of the forum contributors identify how the history of neoliberalism has shaped contemporary authoritarianisation. The victors’ peace constructed following the end of the Cold War certainly played its part. In the post-communist space, the old managerial bureaucracies found an ally in West’s neoliberal agenda, allowing them to create a new oligarchic dominance through rapid privatisation. As the civil society movements of the 1980s in Eastern Europe were not able to generate a critique of Western capitalism that matched their challenge to Soviet authoritarianism, global governance became synonymous with the free flow of capital, not its management and regulation—a return to the disembedded liberalism Polanyi (2001) famously critiqued. For the politicians most associated with the origins and development of neoliberalism, Margaret Thatcher and Ronald Reagan, defeating the left formed part of a moral crusade for a better society. ‘The Reagan Right tended to view politics’, argued Daniel Deudney and G. John Ikenberry shortly after the end of the Cold War, ‘as a war of ideas, an orientation that generated a particularly polemical type of politics’ (Deudney and Ikenberry 1992, p. 133). So, the kind of hyper-partisanship—‘good versus evil’ politics—that today forms a cornerstone of authoritarian protectionism was anticipated by these arguments. But as a defeated left accepted neoliberalism, this, in turn, gave way to its negation: a consensus, eroding the ‘battle of ideas’ necessary for democracy to function (Crouch 2004; Mair 2013).

For Saull, this neoliberal consensus constituted a ‘technocratic and anti-democratic authoritarianism’ that is ‘partly responsible for the far right, populist-authoritarian reaction to it’. Hendrikse has artfully described the new authoritarianism as ‘neo-illiberalism… an amalgam of neoliberal and illiberal operating systems, producing variegated neo-illiberalization across space’ (Hendrikse 2018, p. 70)—and his article in the forum argues that this is ‘not upending but deepening neoliberalism’ and generating the kind of ‘oligarchic class rule’ in the ‘neoliberal heartlands’ that was previously associated with the post-communist world. Chacko’s excellent plotted history of the historical interrelationship between neoliberalism and authoritarianism is theoretically framed by an emphasis on the ‘structural dependence’ of ‘capitalist social orders on extra-economic frameworks’. She highlights the ‘slippage… between advocacy of individual responsibility and family responsibility’ in the work of neoliberal theorist Milton Friedman, who was an acolyte of the Pinochet dictatorship in Chile. Her argument is that this was not a coincidental ‘accident of history’, but indicative of the authoritarian order Friedman was *conceptually* attracted to. A coercive state acts an enforcement mechanism for extra-economic domination through ‘unpaid or law paid social reproduction’ (e.g. by reducing social support for childcare, leaning on tradition for legitimacy), while simultaneously mobilising state power to intensify the exploitation of wage-labour. In short, Friedman’s ego-centric philosophy entailed the reinforcement of patriarchal social organisation and welcomed the construction of fascist-like regimes if they protected and extended opportunities for private capital. My argument here will not challenge this, but emphasise how contemporary authoritarianisation involves a different underlying philosophy.

Chacko rightly notes that the historical construction of capitalism as a social order has always entailed both economic—i.e. the creation of wage-labour markets through the expansion of capital—and extra-economic domination. This contrasts to the Brenner-Wood account of capitalism that holds it generates a distinctively economic logic of exploitation based on capitalist property relations, thus moving away from systems of exploitation based on extraction through ‘political, legal and military coercion’ or ‘traditional bonds or duties’ (Meiksins Wood 1995, p. 29; see also Lacher 2006, pp. 37–38; for a critique see Rioux 2013). As Chacko suggests, the Brenner-Wood position is very problematic. Not only does excluding the extra-economic render the histories of colonialism and slavery conceptually external to capitalism (Anievas and Nişancioğlu 2015, p. 31; Anievas and Nisancioglu 2016), but even ‘the forcible creation and regulation of labour-markets’ required political transformation through, and led by, the state. Thus, this sociological and political process ‘has never been a purely economic phenomenon’ (Banaji 2011, p. 15). I would argue that it follows from such a political reading of capitalism’s rise that what we might call *hegemonic paradigms*—the ideas that give meaning and legitimacy to how power functions in society—will shape the underlying ‘economic’ logics of production (on this see Sayer 1987, pp. 83–112). While these paradigms also existed in the early capitalist period,^3^ they are especially associated with mass politics and the rise of institutions that claimed to be representative of the people (Saull 2015, p. 25). In this sense, what we understand as politics emerged historically as *claims on institutions* that demanded they utilise their extra-economic power to reshape the capitalist order in certain ways.

In the context of this theoretical account of capitalism, we can conclude that both classical neoliberalism as it was pursued in the 1980s and what I have called authoritarian protectionism today constitute hegemonic paradigms.^4^ The aforementioned forum contributors are not wrong to argue that these two frameworks have a number of commonalities. Above all, they are both elite ideologies that involve similar class interests and political traditions—indeed, in some instances, literally the same people that constructed neoliberalism have today shifted to pursue their interests by advancing different ideological reasoning.^5^ Key to this hegemonic shift is a move away from the egoistic individualism that marked the governing project of classical neoliberalism. These sentiments dominated Reagan’s ‘Farewell Address to the Nation’, for example, which described individual freedom as ‘the underlying basis for everything I've tried to do these past 8 years’ and celebrated its global success, even suggesting this had left him surprised (‘We meant to change a nation, and instead, we changed a world’) (Reagan 1989, np). America had been recrafted around the maxim that, as he had earlier put it, ‘government is not the solution to our problem; government is the problem’ (Reagan 1981, np). In short, he argued it was only through unleashing competition between individuals and reducing the state to its core functions that America—and the world—could hope to prosper. Viewed from the conjuncture of 2022 what is striking about such ideas is not only the absence of a *claim to protection* but the explicit refutation of such thinking insofar as it involves the mobilisation of state capacity to secure the wellbeing of the people (‘man is not free unless government is limited’, ‘[a]s government expands, liberty contracts’, etc.) (Reagan 1989, np).

These speeches arguably have a hackneyed, archaic sensibility, because in the dominant ideational frameworks of the contemporary world the ego-centric individual has been displaced by the ethno-nation. Elite politics has moved away from the ‘I won’t protect you’ era of Reagan to the ‘I will’ narrative of Trump. This transformation is perhaps best encapsulated in the Jair Bolsonaro slogan, ‘Brazil above everything, God above everyone’, i.e. the nation is presented as formed through traditional social codes and bonds that entail mutual obligations on the basis of ethno-national kinship. Like other authoritarian protectionists^6^ Bolsonaro’s economic policy has mixed free market^7^ and social welfare^8^ elements. In this vision, merit-based claims are downgraded, and resources become a *right* afforded on the basis of identity and/or in exchange for support. As such, economic inequalities do not tend to be cast as the sum-total outcome of individual self-interest and competition. Thatcher’s successors in Brexit Britain even took up the distributional claims of ‘the left behind’—a terminology that was both entirely alien to the political philosophy of the ‘Iron Lady’ and claimed to be concerned with the persistent, long-term regional inequalities, which are a clear legacy of the 1980s policy of deindustrialisation.

## Authoritarian pathways to the present: the case of the Eurozone crisis

In Saull’s forum contribution, he develops an account of the Eurozone crisis that details how the ‘political economy of neoliberalism’ has enabled ‘the advance of the far-right infused authoritarian protectionism of the present’. While I agree with this basic claim, I have some disagreements with his analyse of the Eurozone crisis. As this is relevant to how we conceptualise the contemporary pattern of authoritarianisation, I will take the opportunity to critique how Saull arrives at this conclusion. At the heart of Saull’s contribution is the claim that the EU is a ‘class project in all but name’, which is committed to an accumulation strategy ‘premised on competition, fiscal consolidation, welfare retrenchment and labour market flexibility’. He argues that ‘the twin-crises of 2008 and 2010’ produced a European crisis regime based upon ‘a highly uneven form of economic development… centred on the relationship between export/credit-surplus economies mainly in the north and typified by Germany and deficit economies in the South typified by Greece and Spain’. Central to his perspective is the combination of an EU technocracy, particularly the monetary authority of the European Central Bank, and a governance structure requiring intergovernmental bargaining. For Saull, the absence of a European demos with sovereign political representation that could give legitimacy to the monetary authority (and the lack of support for such a policy from citizens) locked the Eurozone governance structure into a series of authoritarian pathologies.

While Saull insists that the Eurozone constituted ‘a distinctive kind of uneven and combined development’, it could be argued that this account *is not uneven enough* (for a recent overview of the debates on uneven and combined development see Rosenberg et al. 2022). For the key component of any analysis of the crisis should recognise how convergence around neoliberalism emerged through, and in spite of, the EU’s unevenness. While in crisis conditions the structure of the Eurozone favoured creditor states, this was not a pure ‘beggar-thy-neighbour’ scenario (Dooley 2019) and the category of periphery economy itself contained considerable unevenness (Dooley 2018). So, the dynamics of ideological plurality/cohesion do not map clearly onto the core/periphery categories. EU institutions should also be analysed as polycentric entities that exhibit all the structural and ideational unevenness one would expect given Europe’s complex political geography. The European Commission has occupied a delicate and often contradictory position as both the authority charged with overseeing the neoliberal fiscal rules and an enthusiastic advocate for greater burden-sharing and political integration. In 2012, for example, Commission President José Manuel Barroso put forward a reform vision under the banner of creating a ‘federation of nation-states’ (Barroso 2012). This revisited ‘the concept of political union while stretching its meaning’ (Hodson 2020, p. 301) away from the conception of a ‘pure’ European sovereignty standing over and above member-states. These proposals were also consistent with his predecessor Romano Prodi’s remark of 2001 that the rules of the Eurozone would have to be transformed when they failed in a future crisis.^9^ Germany’s longstanding support for political union likewise provides another point of complexity. This was central to the German justification for austerity enforcement, which they deemed necessary for as long as the EU was not a unitary federal entity (Otero-Iglesias 2017). Germany’s formal support for federalisation is therefore a reminder that the creation of sovereign political representation should not be associated with a particular left or right outcome. In principle, neoliberalism is entirely compatible with creating a federal Europe.

When the crisis struck, the multifaceted nature of the EU’s concrete political alignments (i.e. pro-/anti-austerity not overlapping clearly with pro/anti federalisation), in tandem with the weakness of the anti-austerity left, favoured a neoliberal outcome. Indeed, sometimes in the rush for structural explanations the simple causes are overlooked; chiefly, that the political centre of gravity during the key crisis points went against progressive forces. This is why Saull is wrong when he argues, to ‘all intents and purposes… in the contexts of crisis management the voices of the electorate are not what drives and determines “sovereign” decisions’. While viewed from the narrow vantage point of Greece’s conflict with the Troika in 2015 (where the outcome *did* violate the Greek electorate’s wishes) this is true, even this episode illustrated not the absence of democracy but the problem of reconciling Greek demands with other ‘sovereign decisions’ (Nicolaidis et al. 2018; Nicolaïdis 2013). In this first crisis (2010–2015), the EU’s unevenness as a multi-national bloc was overcome through a neoliberal consensus on the policy framework, which reflected the left’s marginalisation. Indeed, at the time of Syriza’s 2015 rebellion arguably only two Eurozone leaders, François Hollande (France) and Matteo Renzi (Italy), could really be considered to have any kind of electoral mandate opposed to austerity.^10^ What Kalypso Nicolaïdis refers to as the EU’s ‘demoicracy’ character (i.e. drawn from the plural of ‘demos’) (ibid), thus forged the crisis regime due to the inability of reconciling calls for change from some states with the dominant majority. In this sense, we should be analysing the EU/Eurozone as an uneven and combined system, but in terms that draw attention to its holistic sociology, not just economic structure. For its uneven and combined nature is shaped by Europe’s political multiplicity and the form of shared sovereignty integration has generated.

Nonetheless, Saull is right to draw attention to how the Eurozone’s evolution generated strong authoritarian themes, including the role that chauvinistic discourses (‘lazy Greeks’ etc.) played. In this respect, the history of this crisis serves as a reminder both that authoritarianism can be popular, i.e. often embedded in mass, majoritarian sentiments, and that democratic processes can be harnessed for reactionary politics (Mondon and Winter 2020). The crisis revealed a steep failure of European democracy (or its ‘demoicracy’). It exposed how Europe’s rules-based institutions and procedures, i.e. ‘formal democracy’, had built-in authoritarian tendencies due, in part, to the poor design of its post-Maastricht political economy. This, in turn, fed into a substantive democratic failure as austerity undermined the capacity of citizens to have any kind of meaningful control over the forces shaping their lives (Cooper 2021a, pp. 9–10, 67; on the formal and substantive question as it was posed in post-communist Central and Eastern Europe see Kaldor and Vejvoda 2002). The authoritarian surge today—to agree with Saull and others—arises principally from this and other neoliberal failures to establish the stability, wellbeing and security that are necessary for societies to be substantively democratic.

## Neoliberalism, sovereignty and the state: converging and diverging authoritarianisms

Hendrike’s contribution offers a careful review of the book’s arguments. He also questions whether authoritarian protectionism represents a rupture with neoliberalism. ‘Where Cooper emphasises breaches and breaks’, Hendrike argues, ‘he downplays the ways in which neoliberalism is being reproduced via novel alliances and syntheses’. To elaborate this critique, he focuses in on my claim that the Chinese and American patterns of authoritarianisation represent ‘competing models’ (Cooper 2021a, p. 113). While he does not categorically reject this possibility, Hendrikse argues that the ‘key geopolitical and economic story shaping Sino-America is one of convergence’. In the book, I contrast China’s authoritarian model based on high institutional capacity and the subordination of markets and private business to the state with an American version in which public institutions are hollowed out and become an object of rentier claims. In contrast, Hendrikse argues that both the Chinese and US states govern ‘through markets’, and the illiberalism of the Republican Party (seen for instance in the 2022 overturning of Roe vs Wade) is putting America on a path away from competitive electoral democracy. Both the USA and China, he insists, should be understood as ‘corporatist regimes’.

Although these are nuanced disagreements, building a case for Sino-American convergence on an analogous institutional model still seems overstretched. Hendrikse’s review of the book’s COVID-19 chapter, which he generously describes as a ‘brave’ attempt at a first cut analysis of how the pandemic would impact authoritarianisation, is relevant to how we tease out the uneven interactions of Sino-America. In broad agreement with some of my initial analysis, he says that we can ‘identify a clear and remarkable difference in pandemic management between the western Atlantic world and the eastern Asia–Pacific, whereby the latter’s general adherence to zero covid policies proved superior’. Yet, this argument appears to contradict the case he builds for China-US convergence. After all, China’s ‘zero Covid’ strategy was, in part, an expression of its differing institutional form based on the total subordination of markets, society and culture to the Chinese Communist Party (CCP) regime. Xi Jinping’s initial refusal to u-turn, in apparent defiance of internal party concerns,^11^ only to then move dramatically to the other extreme, lifting all restrictions in December 2022, also illustrates the highly personalised form that the dictatorship has assumed under his leadership.

Looking back at the book’s analysis, I did highlight the negative human rights impact of ‘zero Covid’ in China and Vietnam. But the events of 2022 suggest that I was wrong to anticipate that these policies would bring ‘greater global credibility’ to their governance model (Cooper 2021a, pp. 88–89). Vietnam was part of the turn among Asian states away from ‘zero Covid’ in the autumn of 2021. China’s continued commitment to the policy left it isolated internationally as most countries moved towards adaptation and away from elimination. The regime faced mounting problems in the period prior to its abandonment of 'Zero Covid'. Economic growth—for so long the DNA of the CCP’s legitimacy genome—stagnated with the repeated lockdowns. In a rare and perhaps telling sign of public backlash, the regime’s brutal policy of separating children with the virus from their parents in order to place them into state quarantine was dropped (Craft and Reals 2022). COVID-19 showed, in short, that even this most powerful of states is not free of political vulnerability. More broadly, a ‘zero Covid’ policy seems problematic in light of the subsequent evolution of the pandemic. With vaccines and previous infection providing vital immunity but not eliminating the virus from circulation societies are facing up to a long-term and costly (both in terms of economic resources and human wellbeing) adaptation challenge. While, if elimination were possible, it would naturally be desirable, this does not seem to be the case given the virus’ high transmissibility in our globalised world (for an early proponent of adaptation see Meadway 2021a; and also de Waal 2021). In this context, the pursuit of ‘zero Covid’ within individual states is very likely to push towards the extremely undesirable Chinese model based on an ‘unhindered expansion of state control’ (Meadway 2021b).

This COVID-19 example of Sino-American *non-*convergence illustrates the political and institutional unevenness of global authoritarianisation. Nonetheless, Hendrikse is right to identify the broader tendency of big capital to become more enmeshed ‘with political communication, state governance and government’ (see also Hendrikse 2021). This is occurring against a backdrop of weakening economic growth and deepening ecological crisis that incentivise corporations to find new ways to reduce their exposure to risk. As a result, there is a growing importance of sovereign capacities as markets grow ever-more dependent on states. This is seen most clearly in the dramatic mobilisation of state resources during the COVID-19 lockdowns, as the state stepped in to underwrite the huge and uninsurable costs of the shutdowns. But this dynamic also extends beyond ‘the re-emergence of the state as an autonomous actor’ to include ‘the appearance of forms of business organisation [like big tech] with quasi-sovereign capacities’ (Meadway 2021c). Rather than markets expanding as the state retreats (*à la* the claims of neoliberalism), the monopolistic dimension flourishes and becomes intertwined with states. While big corporations may pursue quasi-sovereignty, for many others making rentier claims on existing sovereigns, i.e. states, will offer a more straightforward way of using extra-economic means to mitigate the disruptive effects of heightened systemic risk.

In the face of these structural dynamics, crony capitalism has become a highly prevalent malady. It emerges organically in such a context but may take a variety of forms dependent on how relations between state and capital are organised in specific national geographies. In Hungary, access to financial rents from the state are traded openly in exchange for political patronage, a policy justified as supporting patriotic Hungarian businesses (Miklós and Simons 2021; Szanyi 2022). This is designed to create a semi-democracy with regular elections but an eroded rule of law system, including a de facto un-free media. In China, cognisant of how tech-capitalism has a tendency to monopoly and generating semi-sovereign capacities, the regime seeks its total subordination to one-party rule and a state-managed economic order (Gray and Wang 2022). While Hungary’s model is common, China’s represents a more novel hybrid of communism, ethnic nationalism and tech capitalism. So, in both cases, economic distribution is framed as a right afforded on the basis of ethno-nationality, but this hegemonic paradigm is mobilised in support for divergent political-economic forms (on China’s model see Wong 2022). The overall story, then, is one of simultaneous divergence and convergence as these political experiments occur in a global political economy, facing intensifying ecological and social crises, giving an uneven and combined character to the pattern of authoritarianisation.

## Conclusion: alternatives to authoritarian protectionism?

Structural dynamics—the reassertion of the importance of sovereign capacity in the face of heightened systemic risk—stand behind the transformation between hegemonic paradigms. Classical neoliberal claims that apportion a moral normativity to the functioning of markets free from state interference become unsustainable given these new demands for sovereign capacity and protection (Gerbaudo 2021, chaps 3 and 4). As Mark Blyth and Daniel Driscoll have argued this also raise thorny questions regarding *‘who gets paid off’,* i.e. who is protected, in an energy transition dependent on the mobilisation of massive state resources: big capital demanding state subsidies to maintain profitability or the working and lower classes (Blyth and Driscoll 2022). Authoritarian protectionism offers a hegemonic paradigm attune to these structural dynamics. It is attractive for elites keen to forestall or oppose demands for substantive economic justice and redistribution and connects organically to a historical moment marked by a return of sovereignty.

How do we break free, in this context, from what Hendrikse calls in his forum piece, ‘the authoritarian doom loop’? There are at least three key steps. First, pursue the construction of an alternative ‘claim to protection’ rooted not in ethno-national vocabularies but universal human rights and addressed to the deeply interconnected challenges of socioeconomic and environmental justice. Second, treat the new dependency of markets on states and sovereign capacity as an opportunity to fundamentally retool institutions on democratic lines as vehicles for substantive empowerment, not just formal representation. Third, advance alternatives to extra-economic and economic capitalist logics by expanding domains—that may be territorial or non-territorial (e.g. digital)—in which individuals can associate and cooperate freely. This systemic change agenda needs to be carefully calibrated to ‘the international’ order and its dynamics; for example, recognising the challenge that societies may make antagonistic claims that are equally legitimate from a democratic perspective (on this see Cooper 2021b). These steps may offer some signposts for the long and difficult defence of democracy that lies ahead.

## Footnotes

1 In his book, published contemporaneously to my own Paolo Gerbaudo draws a similar conclusion regarding this new importance of ‘existential threats against which society must be protected’ (Gerbaudo 2021, p. 70).

2 Even Donald Trump’s failed insurrection on January 6, 2021, did not need the critical force that renders Trumpism such a threat to American democracy—his tens of millions of supporters—to be drawn into an active campaign. Instead, it merely required of them to accept the lies he told about the election and his attempted coup.

3 Subjecthood in the sixteenth- and seventeenth-century English-realm-cum-British-empire is one example (Cooper 2022).

4 In the intervening period there was also what Nancy Fraser calls ‘progressive neoliberalism’ (Fraser 2019), i.e., the Blair-Clinton ‘Third Way’, which involved a different set of claims vis-à-vis both classical neoliberalism and authoritarian protectionism.

5 For example, consider the trajectory of Reagan outlier Phyllis Schlafly, who campaigned successfully against the Equal Rights Amendment and whose last pamphlet was titled *The Conservative Case for Trump,* or the Heritage Foundation that played a leading role in the Reagan movement and provided the majority staffers to the Trump White House.

6 The Fidesz government in Hungary and Law and Justice in Poland are both consummate examples of this pragmatic mixing of socially protectionist welfarism (linked to chauvinistic and patriarchal discourses) and neoliberal policy.

7 For example, in 2019 he faced down opposition from the trade unions to pass an austerity-motivated pension reform package (BBC News 2019) and, in the same year, he privatised $23 billion in state assets (MacDonald 2020). Added to this, is his high-profile alliance with sectional business interests that are intent on destroying the Amazon rainforest.

8 He has had difficulty passing his social welfare policy but eventually created the *Auxílio Brasil* programme. In the debate that led to it he claimed he would “never… take from the poor to give to the poorest” (in Uribe et al. 2020).

9 “I am sure the euro will oblige us to introduce a new set of economic policy instruments. It is politically impossible to propose that now. But some day there will be a crisis and new instruments will be created” (in Essex 2011).

10 In 2015, Eurozone governments were dominated by the liberal and right-wing parties, while the few centre-left exceptions mostly supported the anti-Syriza line. Social democrats in Lithuania and Slovakia (the latter government was, in any case, in no way progressive) cited the fact they were poorer than Greece. Malta was a creditor to Greece and its socialist government, which was ensnared in corruption allegations, un-sympathetic. In Austria, the social democrat government maintained the country’s membership of the ‘fiscal hardliners’ group, and in Germany, the centre-left were part of Merkel’s grand coalition. Centre-right governments, even those with economies that could have benefited from the easing of the rules, also took a very tough stance. Pedro Passos Coelho of Portugal, for example, was described by the *Financial Times* as pouring ‘more scorn on the radical aspirations of the Syriza-led government in Athens’ than any other EU leader (Wise 2015).

11 Xi’s threatening statement of May 2022 was interpreted as a sign that there was pushback against ‘zero Covid’ in the party (Gan 2022).
